# The use of respondent‑driven sampling to assess febrile illness treatment-seeking behaviours among forest-goers in Cambodia and Vietnam

**DOI:** 10.1186/s12936-021-04001-9

**Published:** 2021-12-20

**Authors:** Mahesh Paudel, Kemi Tesfazghi, Hoa Nguyen, Sochea Phok, Shwetha Srinivasan, Jennifer Wheeler

**Affiliations:** 1 Population Services International, 1120 19th St NW Suite 600, Washington, DC 20036 USA; 2 Population Services International, VinaFor Building, 127 Lò Đúc, Phạm Đình Hổ Hai Bà Trưng, Hà Nội, Vietnam; 3 Population Services International, 29 St 334, Phnom Penh, Cambodia; 4 Population Services International, T4 Road, Unit 16, Donkoi Village, Sisattanak District, Vientiane Capital, Lao People’s Democratic Republic

## Abstract

**Background:**

Countries in the Greater Mekong sub-region (GMS) aim to eliminate all forms of malaria by 2030. In Cambodia and Vietnam, forest-goers are at an increased risk of malaria. Universal access to prompt diagnosis and treatment is a core malaria intervention. This can only be achieved by understanding the healthcare-seeking behaviour among the most vulnerable groups and eliminating barriers to prompt and effective treatment. This study aimed to explore healthcare-seeking behaviours for febrile illness among populations at risk for malaria in Cambodia and Vietnam.

**Methods:**

In 2019, researchers from Population Services International (PSI) conducted a population-based survey of forest-goers in Cambodia and Vietnam using respondent-driven sampling (RDS) In Cambodia two operational districts, Oral and Phnom Srouch in Kampong Speu Province were included in the study. In Vietnam, communes located within 15 km of the forest edge in Binh Phuoc and Gia Lai Provinces were selected. Adults who had spent at least one night per week or four nights per month in the forest over the previous three months were eligible for the study.

**Results:**

Some 75% of forest-goers in Cambodia and 65% in Vietnam sought treatment for illness outside the home. In Cambodia, 39% sought treatment from the private sector, 32% from community health workers, and 24% from public health facilities. In Vietnam, 62% sought care from community facilities, 29.3% from the private sector, and 6.9% went to a public facility. Among forest-goers who sought care, 33% in Cambodia and 52% in Vietnam did so within 24 h.

**Conclusions:**

This study is consistent with others that show that early diagnosis and treatment of malaria remains an obstacle to malaria elimination. This study also demonstrates that there are gaps in timeliness of care seeking among forest-goers. The findings from this study around provider preference and delays in treatment-seeking can be used to strengthen the design and targeting of malaria interventions and social and behaviour change strategies to accelerate malaria elimination in Cambodia and Vietnam.

**Supplementary Information:**

The online version contains supplementary material available at 10.1186/s12936-021-04001-9.

## Background

Substantial progress has been made toward malaria elimination in the Greater Mekong Sub-region (GMS) where countries are working toward elimination of all forms of malaria by 2030. The number of reported malaria case declined by 86% and the number of malaria-related deaths declined by 97% between 2010 and 2019 [1, 2]. According to the World Health Organization (WHO), the GMS showed a 47% decline in malaria cases from 2019 to 2020. *Plasmodium falciparum* cases no longer account for most cases in the sub-region with *Plasmodium vivax* accounting for majority of the cases. In 2019, *P. falciparum* accounted for only 32% compared to 80% of all cases in 2012. Increased funding and improved vector control, testing and treatment mechanisms have led to a significant decline in cases.

In Cambodia and Vietnam malaria most often occurs in forest and forest-fringe areas. Individuals living or working near the forest have a higher risk of malaria incidence than people who live further from the forest [1]. In Vietnam, approximately 60% of reported malaria cases are among individuals sleeping in forests or on farms [3]. Malaria transmission in Vietnam is highly concentrated in the hilly and forested regions of southern and central provinces along the Laos and Cambodian border where the population is engaged in forest-related activities [4–7]. In Cambodia, a population movement framework (PMF) for malaria was developed [8]. The PMF identified forest workers as having the highest exposure to malaria and lowest access to services, mainly because of their proximity to forests, sub-optimal housing/sleeping conditions [hammocks out in open spaces, in tents or in makeshift shelters], fewer prevention practices, distance from health services, and mobility and lack of engagement with existing community mechanisms for the delivery of interventions or services [8–10].

The distribution of malaria cases in both countries is geographically heterogeneous. Malaria continues to disproportionately affect forest workers, populations living in forest-fringed areas, and migrants. There is substantial population movement within and between countries in the GMS, often driven by poverty, population growth, unemployment, and political oppression [8, 10, 11]. In Cambodia and Vietnam, forest-goers are a key target group for malaria elimination efforts [12].

The WHO Global Technical Strategy calls for ensuring universal access to malaria prevention, diagnosis and treatment as a fundamental pillar of achieving malaria elimination [13]. Despite steep declines in malaria cases, both countries still require significant effort to achieve their malaria elimination goals [2, 4]. Securing access to early diagnosis [usually within 24 h of the onset of symptoms] and treatment for forest-goers is critical [12]. Since the Alma Ata Declaration in 1978, international efforts to improve access to primary health care have focused on strengthening community health worker (CHW) programmes. Community-based interventions by CHWs have successfully improved access to early malaria case detection and treatment [14].

In Vietnam, village health workers (VHWs) are the backbone of the community-level health response. Although not full-time employees of the government, VHWs receive an allowance to engage in outreach activities, which are mainly focused on health promotion and prevention. In Cambodia, the village malaria worker (VMW) initiative was launched by the National Centre for Parasitology, Entomology and Malaria Control (CNM) in 2004 to expand access to early malaria diagnosis and treatment for communities in remote villages where access to health care may be compromised by geographic and economic barriers [15, 16]. In 2009, the VMW cadre was expanded to include mobile malaria workers (MMWs) to target remote populations with active case detection approaches [17].

While efforts to train and deploy health personnel are an important element of ensuring availability of services, ensuring universal access to diagnosis and treatment services can only be achieved by understanding healthcare-seeking behaviours among the most vulnerable groups and by eliminating the barriers they face throughout their healthcare-seeking journeys. In this context, understanding available healthcare-seeking options, perceptions of these services, and healthcare-seeking decision-making is particularly important. The objective of this study was to explore healthcare-seeking journeys for febrile illness among populations at risk for malaria in Cambodia and Vietnam. The study also explored forest-goers’ activities in the forest and key decision-making factors for timely diagnosis and treatment of febrile illness. The results of this study can inform malaria elimination interventions to better reach forest goers most at risk of malaria.

## Methods

A population-based survey of forest-goers in Cambodia and Vietnam using respondent-driven sampling [RDS] was conducted. Due to forest-goers’ hard-to-reach and often mobile status, novel data collection methods were required to gain behavioural insights from this group to inform appropriate malaria elimination interventions. In Cambodia and Vietnam, this group is ‘hidden’ from the general population because of a need for forest-goers to avoid detection by law enforcement officers who regulate forest exploration. RDS is a modified form of snowball or chain referral sampling, which is used to sample hard-to-reach or hidden populations who are linked through social networks in situations where a sampling frame is difficult to obtain [18–21]. This approach uses initial study participants or ‘seeds’ to recruit peers from their social networks. The initial bias created by the non-probability-based selection is addressed using mathematical modelling that allows for inferences to be made about the sampled population [18–21]. Data collection was conducted during high malaria season [22, 23], between 20 and 24 October in Cambodia and between 15 and 26 September in Vietnam.

### Study population

In Cambodia two operational districts, Oral and Phnom Srouch, in Kampong Speu Province were selected. Within these districts, 63 villages were grouped into 21 clusters based on their location and proximity to each other. These districts were selected due to their high incidence of malaria, proximity to the forest, and ease of access to forest-goers. Residents in these forest-fringe areas were considered for the study. In Vietnam, communes located within 15 km of the forest edge in Binh Phuoc and Gia Lai Provinces were selected for the study. Since Vietnam did not have an official definition of forest-goers, for this study forest-goers in Vietnam were defined as people who live inside or within 15 km of the forest and go into the forest at least one night a week or two nights a month.

In both countries, men and women age 18 years and above who spent at least one night per week or four nights per month in the forest over the previous 3 months were considered potential participants for the study. For Cambodia, an additional inclusion criterion, having a fever in the last three months, was used. In Vietnam, individuals with or without fever symptoms in last three months were included in the study. Forest-goers meeting these criteria who produced a coupon or who were selected as a seed were included in the study. Those who did not consent to participate or who did not have a coupon were not included in the study.

### Sampling and forest-goer recruitment

Given the nature of the target population, a clear sampling frame was not available. In both countries, the sample sizes were calculated using a conservative proportion for the main outcome of 50% with a confidence level of 95% and a margin of error of 5%. The design effect was set at 2.0. The resulting required sample size was 675 participants for each country. Sample sizes were calculated separately for forest-goers in Cambodia and Vietnam due to the assumption that the social networks of the forest-goers in the two countries were independent of each other.

**Fig. 1:**
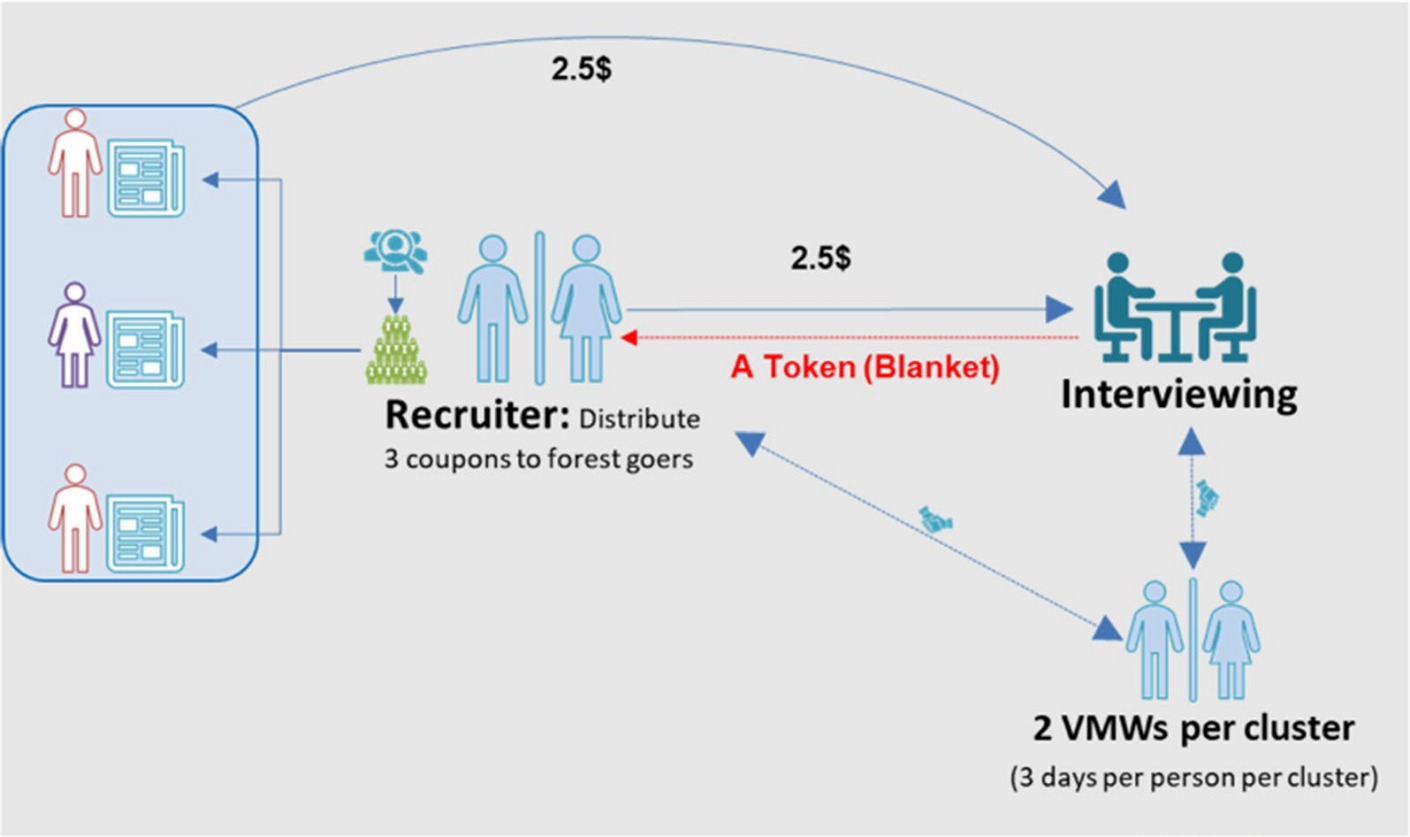
Recruitment and interviewing process

The initial seeds were purposefully selected based on criteria established at the start of the study. The seeds were selected with help from key informants, including community leaders, to ensure that they were representative of forest-goers, were well known, and had a substantial social network. Seeds were provided with uniquely coded coupons (three in Cambodia and five in Vietnam) and asked to recruit subsequent study participants meeting the criteria from within their networks (Fig. 1). The first set of recruits was referred to as the first wave. The first wave was asked to recruit peers as the second wave, and so on, until the estimated sample size was reached.

### Data collection and management

In both countries, the data collection team was divided into four groups with two data collectors per group. The data collectors were trained on the consent process, coupons tracking, incentives management, and the questionnaire and interview process. The data collectors used a tracking form to track the coupons, manage the incentives to the respondents, and track the total number of interviews conducted each day. This form was sent to the field manager, who was responsible for overall recruitment.

The study questionnaire was translated into Khmer and Vietnamese for Cambodia and Vietnam, respectively, and included sections on basic demographics, forest-going frequency and purpose, knowledge and availability of health services within the proximate community, preferences for health service providers, information regarding the most recent fever episode, healthcare-seeking behaviour, and knowledge of malaria transmission. The questionnaire had built-in checks for data quality and was administered using SurveyCTO in Cambodia and SurveyTOGO in Vietnam. The forms were submitted to a central server at the end of each day. Both the research team and field manager conducted onsite data quality checks in addition to online data checks on data collection server.

### Data analysis

Data analysis was performed using the RDS function in Stata 15. Population weights were generated for each variable of interest to control for differences in social network size and homophily of the estimates. The degree (social network size of individual forest-goer) was defined as the number of forest-goers age 18 years and above, that the participant knew who lived in the same community and had spent at least one night per week or four nights per month in forest. Forest-goers were asked about reasons for going to the forest, the number of days they spend in forest, and the last time they were in forest. In addition, all were asked about febrile illness in last three months, whether any treatment was sought for the illness, and the place and time of seeking treatment. Descriptive analyses were conducted for these variables. Chi-square tests were performed to assess the relationship between healthcare-seeking behaviour and demographic variables such as age and gender and malaria risk perceptions. Multivariable logistic regression analyses were conducted to understand associations between preferred type of health facility and reasons for preferences. The dependent variable was the type of facility preferred. The main independent variable was the reason for preferring a specific type of health facility. Stepwise removal of independent variables (p > 0.05) was used to derive the final model. Each reason for preference was dummy coded as a separate variable for the analysis. Individuals with missing data were excluded from the analysis. Age and education were included as confounding variables in the model. All analyses were performed separately for the two countries, as networks in each country were assumed to be independent of each other. Furthermore, inclusion criteria for two countries were slightly different; fever in the last three months was an inclusion criterion in Cambodia but not in Vietnam.

### Ethical consideration

The study design and protocols for the study were reviewed and approved by the PSI Ethics Board, the National Ethics Committee for Health Research for Cambodia, and the Hanoi School of Public Health Ethics Board for Vietnam. Informed consent was obtained from participants prior to enrolment in the study.

## Results

### Recruitment

Among Cambodian forest-goers, 21 initial seeds recruited 654 study participants over 3–6 waves of recruitment. In Vietnam, 15 initial seeds recruited 633 study participants over 3–5 waves of recruitment. In both countries 90% of coupons were returned. Participation was voluntary, and those who received a coupon from a peer but were not interested in the study had no obligation to contact the study team.

All participants had spent a night in forest in the last week or at least four nights per month in the last 3 months. Most participants were recruited by their friends (96.9% in Cambodia and 92.3% in Vietnam) and had known the person who had recruited them for more than a year (91.6% in Cambodia and 79.3% in Vietnam). The forest-goers in Cambodia reported knowing fewer other forest-goers compared to those in Vietnam, with 40% of forest-goers in Cambodia knowing five or fewer and 48.9% of forest-goers in Vietnam knowing 10 or more other forest-goers. Table 1 shows the unweighted analysis of recruitment patterns in Cambodia and Vietnam.

**Table 1:** Unweighted analysis of recruitment patterns

	Cambodia	Vietnam
	Unweighted proportion [N = 675]	Unweighted proportion [N = 648]
Reasons for participation		
Interested in study topic	57.8	28.2
Wanted to help the community	14.1	59.7
My friend wanted me to participate	25.0	1.4
Incentive/gift	3.0	10.7
Others	0.2	–
Known the person		
< 6 months	4.4	11.4
6–12 months	0.9	1.9
More than a year	91.6	79.3
Missing	3.1	7.4
Number of other forest-goers known		
Five or less	40.0	26.1
6–10	34.5	24.1
More than 10	25.5	48.9
Don’t know	–	0.9
Mean	12.04	21.04

### Demographics

Most of the forest-goers in Cambodia were married (84.3%), male (85.5%), under 40 years old (73.2%), and had completed primary education (47.8%). Almost 80% of forest-goers earned their living through forest-related work but made very little, with 95.4% earning less than US$500 per month and 18.3% reporting being part of an Identification of Poor (IDPoor) household. IDPoor is a mechanism developed by Cambodia Ministry of Planning to identify poor and vulnerable households for development programmes in Cambodia [24].

In Vietnam, most of the sampled forest-goers were married (84.8%), male (95.2%), and between the ages of 25 and 34 years (52.5%). In Vietnam, forest-goers under 35 years constituted over 70% of all forest-goers sampled, while the age distribution in the Cambodian forest-goers was more evenly spread across the age groups. The age of forest-goers ranged from 18 to 70 years in Cambodia, while in Vietnam age ranged from 20 to 60 years. Most forest-goers in Vietnam made their living through agriculture, and like Cambodia, almost all (99.7%) made less than US$500 per month.

Forest-goers in Vietnam were long-standing residents of their villages, with a majority (96.6%) having lived there for more than 10 years. In Cambodia, there was variation in duration of residence, with 25.5% of residents living in their village less than 10 years and 29.2% living there for 30 years or more. Table 2 shows the weighted demographic characteristics of forest-goers in Cambodia and Vietnam.

**Table 2:** Weighted demographic characteristics of forest-goers

	Cambodia		Vietnam	
	Weighted population proportion [Bootstrapped 95% CI]	Unweighted sample, N	Weighted population proportion [Bootstrapped 95% CI]	Unweighted sample, N
Gender				
Male	85.5 [80.9–89.1]	591	95.2 [91.5–98.0]	633
Female	14.5[10.9–19.1]	84	4.8 [2.0–8.5]	15
Age [years]				
< 25	20.1 [16.3–24.2]	133	21.2 [16.4–25.9]	120
25–29	18.6 [15.2–22.3]	127	23.0 [18.5–27.5]	162
30–34	17.1 [13.5–20.8]	115	29.5 [24.3–34.6]	196
35–39	17.4 [13.8–21.0]	120	13.8 [8.7–18.9]	88
40+	26.7 [22.2–31.5]	180	12.6 [9.3–15.8]	82
Education				
Never attended school	14.4 [11.2–18.1]	100	6.0 [3.8–8.4]	38
Attended school, but never completed	24.8 [20.8–29.4]	198	35.5 [28.5–42.7]	148
Primary school	47.8 [42.5–52.7]	300	12.9 [9.6–16.3]	96
Lower Secondary school	11.7 [8.6–15.0]	70	11.6 [8.5–15.3]	121
Upper Secondary or Higher	1.3 [0.4–2.4]	7	13.0 [10.1–16.3]	140
No response	–	–	21.1 [13.8–302,]	105
Income				
< $500	95.4 [93.6–97.1]	637	99.7 [99.1–99.9]	640
> $500	3.9 [2.4–5.5]	33	0.3 [.05-.9]	8
Don’t know	0.8 [0.1–1.7]	5	–	–
ID Poor^a^	18.3 [14.1–22.5]	117	–	–
Occupation				
Agriculture	16.3 [13.0–20.0]	125	58.3 [51.1–64.3]	389
Forest-related	77.9 [73.6–81.8]	507	26.9 [21.6–32.9]	165
Others	5.7 [3.6–8.6]	43	15.0 [11.1–19.7]	94
Marital status				
Currently married	84.3 [80.5–87.7]	568	84.8 [79.8–89.2]	571
Single or formerly married	15.7 [12.3–19.5]	107	15.2 [10.8–20.2]	77
Time at place of residence				
10 years or less	25.3 [20.8–30.1]	156	3.4 [1.8–5.8]	39
11–20 years	26.9 [22.6–31.6]	184	8.6 [5.7–11.7]	79
21–30 years	27.9 [23.4–32.5]	199	44.5 [38.7–50.5]	267
More than 30 years	29.9 [15.6–24.5]	135	43.5 [37.8–49.2]	263

^a^IDPoor is a mechanism developed by Cambodia Ministry of planning to identify poor and vulnerable households for development programs in Cambodia

### Forest-going and activities in the forest

The primary reason for going to the forest was cutting wood (85%) for Cambodian forest-goers while only 17.9% went for wood in Vietnam. Bamboo (15.4%) and charcoal (14.7%) were other reasons for going to the forest in Cambodia. In Vietnam, plantation work was the main reason for going to forest: 28% worked in cashew plantations, 27.7% in wheat plantations, and 19.4% in rubber plantations. In Cambodia, 78.4% went to the forest for one reason only and 18% went for two or more reasons. Forest-goers in Vietnam reported spending more nights in the forest in the last 30 days compared to forest-goers in Cambodia. Over two-thirds (71.1%) of forest-goers in Cambodia and more than half (54.2%) in Vietnam reported spending seven nights or fewer in the forest in the last 30 days. Forest-goers in Vietnam reported spending a longer time in the forest, with 23.6% in Vietnam spending 8–14 nights and 22.3% spending 15 nights or more in the forest in the last 30 days. Comparatively, only 8.5% of forest-goers in Cambodia spent 15 nights or more in the forest. The interval between forest visits was longer in Cambodia. Almost one-fifth (19.8%) of forest-goers in Cambodia had an interval of 15 or more days since their most recent forest visit compared to 14% in Vietnam. Most forest-goers in Cambodia and Vietnam reported having been to the forest in the last seven days (69.8 and 76.6%, respectively) (Table 3).

**Table 3:** Weighted forest-going and activities in forest

	Cambodia		Vietnam	
	Weighted population proportion [Bootstrapped 95% CI]	Unweighted sample, N	Weighted population proportion [Bootstrapped 95% CI]	Unweighted sample, N
Reasons for going to the forest^a^				
Wood	85.1 [80.9–88.8]	675	17.9 [14.3–22.1]	648
Charcoal	14.7 [10.3–20.1]	675	–	–
Bamboo	15.4 [10.7–21.0]	675	–	–
Fishing	–	–	14.5 [11.4–17.9]	648
Rubber plantation	–	–	19.4 [14.1–25.6]	648
Cashew plantation	–	–	28.0 [21.8–34.4]	648
Wheat plantation	–	–	27.7 [22.4–33.3]	648
Others	7.5 [5.3–10.3]	675	35.5 [28.7–42.8]	648
Number of reasons for going to forest				
One	78.4 [73.9–82.7]	524	49.7 [43.8–55.6]	292
Two	18.6 [14.6–22.7]	123	41.7 [35.9–47.4]	250
Three or more	3.0 [1.6–4.6]	28	8.6 [6.0–11.2]	106
Nights spent in forest in last 30 days				
Short [≤ 7 days]	71.1 [66.8–74.8]	434	54.2 [47.5–60.8]	401
Medium [8–14 days]	20.5 [16.9–24.3]	161	23.6 [18.6–29.0]	138
Long [15 + days]	8.5 [6.4–10.9]	80	22.3 [16.7–28.3]	109
Last time in forest				
7 days or less	69.8 [65.0–74.7]	471	76.6 [71.1–82.1]	471
8–14 days	10.4 [7.6–13.1]	71	9.4 [4.8–14.0]	56
15 + days	19.8 [15.9–24.0]	133	14.0 [10.4–17.6]	121

^a^Multiple response question

### Healthcare-seeking behaviour

All respondents from Cambodia and 65.8% of respondents from Vietnam reported having febrile illness in the last three months. More than half (52.2%) of respondents in Cambodia reported suspecting that their febrile illness was malaria, 13.4% had a cold, 10.1% had *Krunkdov/kdovkhlounr* (general fever), and 9.5% had typhoid. In Vietnam, 41.6% of the cases were flu, followed by malaria (30.1%), colds (15.5%), and other febrile illnesses (11.9%). Less than half (42.9%) of forest-goers in Vietnam had a blood test following the febrile illnesses and almost all who did (94.5%) visited a public health or community health facility for the blood test. In Cambodia, 66.5% of the forest-goers went for a blood test with 36.7% seeking testing from CHWs, followed by private health facilities (33.6%) and public health facilities, mostly health centres (28.0%). In Vietnam, 73.1% of those who received a blood test tested positive for malaria, while 62.5% tested positive for malaria in Cambodia.

Most forest-goers in both countries sought treatment for illness. Three-quarters (75%) of the forest-goers in Cambodia sought treatment outside of their home for febrile illness, 17.4% did not seek any treatment and 7.6% were treated in their home. In Vietnam, 65.3% of those with febrile illness sought treatment outside of their home and 7.3% sought treatment at their home. However, more than a quarter (27.4%) of those with febrile illness did not seek any treatment. Bivariable analysis showed that seeking treatment from any source was significantly associated with age (χ^2^ = 9.7, *p* < 0.001) and gender (χ^2^ = 27.3, *p* < 0.001) for Vietnam while results were not significant for Cambodia.

In Cambodia, 39% of those seeking care for febrile illness first sought treatment from the private sector, followed by CHWs (32.4%) and public health facilities (24.1%). In Vietnam, 62.3% sought care from community facilities, 29.3% from the private sector, and only 6.9% from a public facility. The comparison between place of blood test and first place of treatment suggests that some forest-goers in Vietnam opted to go to the private sector after getting a blood test in the public sector. The first place of treatment was significantly associated with age (χ^2^ = 2.4, *p* < 0.05) and perceived malaria infection (χ^2^ = 9.0, *p* < 0.001) in Cambodia. Associations were not significant for Vietnam. A similar shift between CHWs and private health facilities was observed in Cambodia. Among Cambodian forest-goers who sought care, 33.1% did so within 24 h, 38.4% sought care within 24–48 h and 28.5% waited for three or more days before seeking care for the febrile illness. In Vietnam, more than half (51.6%) of those who sought care for febrile illness did so within 24 h, 13.4% sought care in 24–48 h and 35% sought care after 48 h. Time to seeking treatment was associated with age (χ^2^ = 3.4, *p* < 0.01) in Cambodia. Results were not significant for gender or education.

Some 69.4% of the forest-goers in Cambodia sought treatment for their illness from one place while 30.6% went to two or more places. In Vietnam, a majority (58.2%) of forest-goers visited more than one place. Table 4 describes key healthcare-seeking behaviours of forest-goers in Cambodia and Vietnam.

**Table 4:** Healthcare-seeking behaviour of forest-goers

	Cambodia		Vietnam	
	Weighted population proportion [Bootstrapped 95% CI]	Unweighted sample N	Weighted population proportion [Bootstrapped 95% CI]	Unweighted sample N
Fever in last 3 months				
Yes	100	675	65.8 [60.0–71.3]	427
Kind of febrile illness suspected by forest goers			*[n* = *427]*	
Malaria	52.2 [47.3–57.2]	334	30.1 [23.1–38.1]	124
Dengue	4.8 [2.9–7.0]	29	1.0 [0.2–2.2]	6
Typhoid	9.5 [7.0–12.3]	70	–	
Cold	13.4 [10.0–17.3]	97	15.5 [10.8–21.0]	115
Flu	2.8 [1.4–4.2]	17	41.6 [33.4–50.1]	155
*Krunkdov/kdovkhlounr* [general fever]	10.1 [7.4–13.2]	79	–	
Other/don’t know	7.3 [5.0 -9.8]	51	11.9 [4.6–19.6]	27
Blood testing			*[n* = *427]*	
Had blood tested	66.5 [61.8–71.7]	459	42.9 [34.8 -51.2]	199
Place of blood testing	*[n* = *459]*		*[n* = *199]*	
Public health facility	28.0 [20.4–36.7]	105	94.5 [89.7–98.2]	175
Private health facility [includes pharmacy/chemist/drug shop]	33.8 [26.5–41.2]	181	n/a	n/a
Community health worker	36.7 [27.9–45.8]	162	–	–
Others	1.5 [0.5–2.6]	11	5.5 [1.8–10.3]	24
Result of blood test	*[n* = *459]*		*[n* = *199]*	
Positive for malaria	62.5 [55.4–69.7]	253	73.1 [54.7–90.3]	116
Negative/inconclusive for malaria	21.2 [15.7–27.0]	114	20.3 [3.2–39.5]	12
Non-malaria diagnosis	15.5 [10.2–21.4]	85	6.6 [2.1–12.6]	71
No response	0.8 [0.1–2.4]	7	–	–
Sought treatment for illness from any source			*[n* = *427]*	
Sought treatment from any source	82.6 [78.9–85.9]	526	67.8 [59.4–75.4]	303
Sought treatment for illness outside of home			*[n* = *427]*	
Sought treatment outside home	75.0 [70.9–79.2]	481	65.3 [57.0–72.9]	279
First place sought treatment from	*[n* = *526]*		*[n* = *303]*	
Public health facility	24.1 [17.9–30.6]	111	6.9 [3.7–10.5]	21
Private health facility [includes pharmacy/chemist/drug shop]	39.0 [32.2–45.6]	252	29.3 [18.9–40.2]	81
Community health Worker	32.4 [25.9–39.4]	138	–	–
Community health Facility	–	–	62.3 [51.4–72.5]	187
Other	4.6 [2.6–6.9]	25	1.5 [0.3–4.1]	14
Time to seek treatment	*[n* = *526]*		*[n* = *303]*	
≤ 24 h [0–1 days]	33.1 [27.2–39.3]	174	51.6 [36.0–65.8]	81
48 h [2 days]	38.4 [32.7–44.2]	205	13.4 [8.0–20.2]	75
> 48 h [≥ 3 days]	28.5 [22.9–34.4]	146	35.0 [23.4–48.4]	147
Number of places sought treatment from	*[n* = *525]*		*[n* = *303]*	
One place	69.4 [63.6–74.6]	380	41.8 [33.0–50.2]	172
More than one	30.6 [25.4–36.3]	145	58.2 [49.8–67.0]	260
Second place of treatment	*[n* = *525]*			
Public health facility	6.0 [3.7–9.0]	30	n/a	n/a
Private health facility [includes pharmacy, chemist, and drug shop]	22.0 [17.1–27.5]	104	n.a	n.a
Others	2.5 [0.9–4.6]	11	n/a	n/a
Sought treatment at one place only	69.4 [63.1–74.5]	380	n/a	n/a

Krunkdov/kdovkhlounr is local term for general fever. Sub-sample (*n*) is presented in italics

### Preferred place of treatment

Among forest-goers in Cambodia, private health facilities (41.2%) were the most preferred place of treatment for febrile illness, followed by CHWs (31.4%) and public health facilities (25.3%) (Table 5). In Vietnam, 44.5% preferred community health facilities, followed by private health facilities (39.3%) and public health facilities (10.8%). Table 5 describes forest-goers’ preferred place of treatment in Cambodia and Vietnam. In both countries, preferred place of treatment was significantly associated with age of forest-goers (Cambodia: χ^2^ = 3.3, *p* < 0.05; Vietnam: χ^2^ = 12.5, *p* < 0.001). The results were not significant for education, income and gender.

**Table 5:** Preferred place of treatment

	Cambodia		Vietnam	
	Weighted population proportion [Bootstrapped 95% CI]	Unweighted sample N	Weighted population proportion [Bootstrapped 95% CI]	Unweighted sample N
Preferred place of treatment				
Public health facility	25.3 [20.3–30.7]	155	10.8 [7.9–13.9]	97
Private health facility [includes pharmacy, chemist, and drug shop]	41.2 [35.7–46.7]	337	39.3 [32.6–46.2]	208
Community health worker	31.4 [25.6–37.6]	168	–	–
Community health facility	–	–	44.5 [38.2–50.9]	308
Other	2.1 [0.8–3.7]	15	5.4 [3.3–8.1]	35

Forest-goers were asked to rank their top three reasons for preferring a particular health facility. Forest goers in Cambodia reported a mix of reasons for preferring a particular facility (Table 6). Proximity (37.7%), quality of service (16.3%) and trust in provider (15.2%) were some of the ranked top reasons for preferring a facility in Cambodia. Forest-goers in Vietnam were asked to rank their reasons for health facility preferences; this resulted in more clearly articulated drivers of provider preferences. Perceived proximity to provider (87.8% of forest-goers) was the top ranked reason for facility choice. Cost (68% of forest-goers) was the second ranked and availability of services (45.8%) a third top reason for choice of facility. Quality of services and trust in provider were not as frequently ranked as reasons for preferring a facility in Vietnam as in Cambodia.

**Table 6:** Reasons for preference

	Cambodia		Vietnam	
	Weighted population proportion [Bootstrapped 95% CI]	Unweighted sample N	Weighted population proportion [Bootstrapped 95% CI]	Unweighted sample N
Top ranked reasons for place of preference				
Proximity	37.7 [32.7–43.1]	239	87.8 [84.2–91.0]	549
Second ranked reasons for place of preference				
Cost	36.5 [31.7–41.2]	208	68.0 [62.6–73.4]	417
Third ranked reasons for place of preference				
Availability of service	–	–	45.8 [39.4–52.2]	168
Trust in provider	38.8 [32.8–44.9]	194	16.1 [12.4–20.0]	131

[Refer to Additional file 1: Table S1 for complete table]

In Cambodia, cost (OR = 5.16, *p* < 0.001) quality of service (OR = 2.28, p < 0.001), friendliness of service (OR = 2.29, p < 0.001) and availability of services (OR = 6.26, p < 0.001) were positively associated with the preference of public health facilities while trust in provider (OR = 0.36, p < 0.001 was negatively associated with public health facility preference (Table 7). The F-value of the model was 13.29 (p < 0.001), which implies that the independent variables significantly predict the dependent variable.

**Table 7:** Weighted odds ratios for the reasons for facility preference

Cambodia	Odds Ratio	p-value
Public facility		
Cost	5.16	< 0.001
Quality of service	2.28	< 0.001
Friendliness of service	2.29	< 0.001
Availability of service	6.26	< 0.001
Trust in provider	0.36	< 0.001
Private facility		
Proximity	0.33	< 0.001
Cost	0.23	< 0.001
Recommended provider	0.13	0.001
Availability of service	0.35	0.001
Trust in provider	1.74	0.003
Others	4.00	0.012
Community Health Workers		
Proximity	4.80	< 0.001
Recommended provider	9.22	0.001
Quality of service	0.34	< 0.001
Friendliness of service	0.37	0.001
Trust in provider	1.65	0.034

Vietnam	Odds Ratio	p-value
Public facility		
Proximity	0.01	< 0.001
Cost	0.06	< 0.001
Previous experience	0.03	< 0.001
Recommended provider	0.06	< 0.001
Friendliness of service	0.01	< 0.001
Availability of service	0.01	< 0.001
Trust in provider	0.02	< 0.001
Private facility		
Proximity	0.17	< 0.001
Cost	0.57	0.014
Quality of service	0.15	< 0.001
Friendliness of service	0.16	< 0.001
Availability of service	3.04	< 0.001
Community Health Facility		
Cost	0.12	< 0.001
Previous experience	0.09	< 0.001
Recommended provider	0.08	< 0.001
Quality of service	0.01	< 0.001
Friendliness of service	0.25	0.007
Availability of service	0.02	< 0.001
Trust in provider	0.06	< 0.001

Trust in provider (OR = 1.74, p < 0.05) and other reasons (OR = 4.00, p < 0.05) were positively associated with preference of private facilities. Cost (OR = 0.23, p < 0.001), proximity (OR = 0.33, p < 0.001), recommended provider (OR = 0.13, p < 0.01) and availability of services were negatively associated with preference of private facilities in Cambodia. (F-value = 13.11, p < 0.001).

Proximity (OR = 4.80, p < 0.01), recommended provider (OR = 9.22, p < 0.001) and trust in provider (OR = 1.65, p < 0.05) were associated positively with preference of CHWs in Cambodia. Quality of services and friendliness of service were negatively associated with preference of CHWs. The F-value of the model was 11.48 (p < 0.001).

In Vietnam, only availability of services (OR = 3.04, p < 0.001) was positively associated with preference of private health facilities. Other factors: proximity, cost, quality of service and friendliness of service, were negatively associated with preference of private providers. The F-value of the model was 18.75 (p < 0.001). Although community health facilities were the preferred place of almost half of forest-goers, none of the factors were positively associated with preference for this type of facility (F-value = 18.1, p < 0.001). Quality of services was not included in the public health facility analysis because it was rarely ranked as a reason for preference. Upon dropping this variable, the remaining factors were not positively associated with a preference for public health facilities (F-value = 16.1, p < 0.001).

## Discussion

In both countries most of the forest-goers were married men. Between 50 and 60% of forest-goers were under 35 years old in Vietnam and Cambodia. Forest-goers in Vietnam spent more nights in the forest in the last 30 days compared to forest-goers in Cambodia. Findings from this study are consistent with studies that have explored the behaviours of forest-goers in Vietnam and mobile migrant populations (MMPs) in western Cambodia, which found that forest-goers spend extended periods in the forest, sleep in temporary structures and do not routinely use long-lasting insecticidal nets (LLINs) resulting in increased risk of malaria [25].

### Care-seeking behaviours amongst forest-goers

This study is consistent with others that show that early diagnosis and treatment of malaria remain obstacles to malaria control and elimination activities. In Cambodia and Vietnam the national guidance is for all suspected malaria patients to receive a parasitological test [26]. However, this study demonstrates that among those most at risk of malaria, i.e., forest-goers in Cambodia, 25% either did not seek treatment at all or were treated at home (17.4 and 7.6%, respectively). This study also demonstrates that there are gaps in timeliness of care seeking among forest-goers. In Vietnam only 33% of forest-goers sought care within the 24-h period recommended by WHO, whereas 38.4% sought care within 24–48 h and 28.5% did not seek care for more than 3 days. Among Cambodian forest-goers who sought care, only about one-third did so within 24 h. Prior qualitative research provides further context for these study findings. These studies describe health-seeking journeys in the GMS context, where economic pressures drive forest activities and indicate that efforts to protect work time and corresponding income have a strong impact on decisions to abandon economic activities in the interest of seeking medical attention. These studies have found that an individual’s healthcare-seeking behaviour is not a static construct but is influenced by the individual’s classification of the illness [27]. Classification of an illness as ‘small’ or ‘big’ impacts the healthcare-seeking journey for that episode [15]. Ultimately, the perceived severity of an illness has an impact on decisions regarding if and when to seek care. Ironically, the combined impact of significant reductions in malaria incidence and mortality combined with increased access to healthcare at village level in both Vietnam and Cambodia has lessened the perceived severity of the disease and may compromise the perceived need to seek care promptly [27]. Furthermore, forest-goers often begin self-treatment when they are in the forest or before they go into the forest [5, 28].

### Preferred source of care

All respondents in Cambodia [as part of the inclusion criteria] and more than 60% in Vietnam reported febrile illness in last three months. Of those reporting febrile illness, one in two in Cambodia and one-third in Vietnam mentioned that they had malaria. The majority of those seeking treatment for febrile illness in Cambodia sought private health care. This finding is consistent with a prior study from western Cambodia in which more than half of study participants sought care from the private sector [25]. Similarly, in a study conducted in the Bago region of Myanmar, migrant workers preferred to seek care from the private or informal healthcare sector because of convenience, trust and availability of low-cost anti-malarial medication [29]. In a study previously conducted in Kampot, Cambodia, a majority of forest-goers sought care in the private sector and cited factors influencing care-seeking behaviour that included proximity, reputation of the provider, trust, and perceived severity [15].

A review of qualitative literature from the Mekong region showed that despite free diagnosis and treatment services in the public sector, forest-goers often sought services in the private sector due to service quality and accessibility [10]. This apparent preference for the private sector is in contrast to the findings in this study in which a higher proportion of respondents in Vietnam sought care from community health facilities and only one-third sought care from the private sector [5]. These findings may be explained by the fact that Vietnam has a well-established public healthcare network that extends to the commune level and includes a strong community-level health service. Anthropological studies in Vietnam also report strong confidence in the public health system at the community level [6]. However, concerns around gaps in coverage of the public health system for migrants have been observed in situations when national identification is required to access care, making private sector services a necessary option for care [5].

Community-based interventions are often a first source of treatment for malaria in remote areas where access to formal health services is limited [30]. In Cambodia, however, VMWs specialize only in malaria prevention and case management, which likely explains why they were not usually the first point of care among forest-goers in this study [15]. Furthermore, community volunteers often juggle service provision with paid employment and can be perceived as an unreliable service option due to unavailability in remote areas [10]. Just over 7% of forest-goers in both countries sought treatment at home in this study, consistent with previous studies that have identified the use of an array of treatment options, including traditional methods and over-the-counter drugs from pharmacies and other vendors for self-treatment [10].

The comparison between place of blood test and first place of treatment suggests that some forest-goers opt to go to the private sector after getting a blood test in the public sector; similar transitions between provider types were observed in Cambodia where patients shifted between using CHWs and the private sector. This use of multiple types of providers appears to be more prominent in Vietnam than in Cambodia, where a majority of forest-goers sought treatment from one place; in Vietnam, the majority visited more than one place. These findings suggest that provider preference is not static, but is influenced along the journey of the patient’s experience.

This study shows that cost, quality of service, friendliness of service, and availability of services are key factors for choosing a public health facility in Cambodia. In a study conducted in rural Cambodia, villagers provided different reasons for trusting public and private providers. Public providers were trusted because of their abilities and the presence of an effective referral system. On the other hand, private providers were trusted because they were friendly, thorough and easy to contact [31]. However, in this study, trust in provider was negatively associated with preferring a public provider but positively associated with preferring a private facility. In Vietnam, cost, recommended provider and quality of service were positively associated with preferring a public health facility while only availability of services was positively associated with preferring a private health facility. Despite being the preferred place for almost half of the forest-goers, none of the factors measured in this study was positively associated with a preference for community health facilities.

## Limitations

The data in this study were self-reported by participants and do not include observational behavioural data or data from health facilities. Some recall bias is therefore possible. In addition, social desirability could also have influenced some of the responses. Additionally, there was no attempt to calculate the network size in either of the study areas, and this information is not included in the study results. Finally, the two samples were treated as independent networks and thus comparative analyses between the two countries were performed. In addition there may be other factors associated with facility preference that were not captured by this study, which could have impacted the results.

## Conclusion

Forest-going remains a major risk factor for malaria in Cambodia and Vietnam; however, economic pressures compel people to undertake this activity. With the rapid decline of malaria in both countries, malaria has become more concentrated geographically and among the most at risk populations. Studies using respondent driven sampling are a powerful means of gathering insights into the behaviour of forest-goers who are otherwise difficult to access. The findings from this study about provider preference and delays in treatment-seeking behaviour can be used to strengthen the design and targeting of malaria interventions, as well as social and behaviour change strategies to accelerate malaria elimination in Cambodia and Vietnam.

## Supplementary Information

**Additional file 1: Table S1. **Reasons for preference**.**

## Data Availability

The datasets used and analysed during the current study are available from the corresponding author on reasonable request.
