# Supplementary material for: The use of respondent‑driven sampling to assess febrile illness treatment-seeking behaviours among forest-goers in Cambodia and Vietnam
Source: Malar J. 2021 Dec 20;20:477. doi: 10.1186/s12936-021-04001-9 (PMC8686608; doi:10.1186/s12936-021-04001-9)
Supplement: Supplementary file 1 — Additional file 1: Table S1. Reasons for preference. [file 12936_2021_4001_MOESM1_ESM.docx]

**Table S1 Reasons for preference**

|  | **Cambodia** | | **Vietnam** | |
| --- | --- | --- | --- | --- |
|  | **Weighted population proportion [Bootstrapped 95% CI]** | **Unweighted sample N** | **Weighted population proportion [Bootstrapped 95% CI]** | **Unweighted sample N** |
| **Top ranked reasons for place of preference** |  |  |  |  |
| Proximity | 37.7 [32.7-43.1] | 239 | 87.8 [84.2-91.0] | 549 |
| Cost | 14.6 [11.3-18.0] | 97 | 4.7 [3.0-6.8] | 38 |
| Previous experience | 7.8 [5.3-10.4] | 56 | 3.4 [1.8-5.2] | 32 |
| Recommended provider | - | - | 1.3 [0.5-2.3] | 18 |
| Quality of Service | 16.3 [12.6-20.4] | 102 | 1.9 [0.4- 4.0] | 6 |
| Friendliness of service | 5.4 [3.4-7.6] | 38 | 0.8 [0.1-2.4] | 5 |
| Trust in provider | 15.2 [11.9-18.8] | 121 | - | - |
| Others | 3.2 [1.8-4.8] | 22 | - | - |
| **Second ranked reasons for place of preference** |  |  |  |  |
| Proximity | 10.1 [7.2-13.3] | 66 | - | - |
| Cost | 36.5 [31.7-41.2] | 208 | 68.0 [62.6-73.4] | 417 |
| Previous experience | 6.0 [3.8-8.7] | 44 | 16.3 [12.2-20.6] | 107 |
| Recommended provider | - | - | 4.7 [2.9-7.0] | 32 |
| Quality of Service | 15.8 [12.4-19.7] | 96 | 6.3 [4.4-8.5] | 72 |
| Friendliness of service | 10.0 [7.0-13.0] | 56 | 1.9 [0.4-3.8] | 6 |
| Availability of service | - |  | 2.8 [1.2-5.2] | 14 |
| Trust in provider | 16.8 [13.0-21.1] | 112 | - | - |
| Others | 4.7 [2.7-7.0] | 30 | - | - |
| **Third ranked reasons for place of preference** |  |  |  |  |
| Proximity | 11.1 [7.9-14.7] | 64 | - | - |
| Cost | 18.1 [14.2-22.3] | 95 | - | - |
| Previous experience | 5.4 [3.1-8.4] | 34 | 16.5 [12.5-20.8] | 163 |
| Recommended provider | - | - | 2.5 [1.4-3.8] | 28 |
| Quality of Service | 11.6 [8.2-15.2] | 68 | 5.6 [3.5-7.9] | 52 |
| Friendliness of service | 10.3 [7.0-13.9] | 64 | 13.4 [10.3-16.8] | 102 |
| Availability of service | - | - | 45.8 [39.4-52.2] | 168 |
| Trust in provider | 38.8 [32.8-44.9] | 194 | 16.1 [12.4-20.0] | 131 |
| Others | 4.7 [2.2-8.3] | 27 | 0 | 4 |
